# Excisions of Enlarged Tonsils

**Published:** 1840

**Authors:** 


*Enlargement of the Tonsils—Excision—Cure.*—M. J. Rodgers, ætat, 16, enjoying apparently robust health, was admitted, July 14th, for enlargement of the tonsils. She states that for several years past she has been subject to repeated attacks of sore throat—has for a long time observed that her enunciation was constantly becoming more difficult, and has a constant uneasy sensation in her throat, and frequently, when in the recumbent position, difficulty of respiration. Upon examination, the tonsils were found to be considerably enlarged, with a general redness of the back part of the throat. On the day after entering the house, the left tonsil was excised by means of the sector tonsillarum of Dr. Fahnestock. Six days afterwards, the tonsil of the right side was removed. The hæmorrhage resulting from these operations was trifling, and the slight soreness of throat which followed was entirely removed after a few days. She was discharged cured, August 24th.

